# Distal femoral replacement for a periprosthetic fracture: a method for removing the distal fragment

**DOI:** 10.1308/003588412X13373405387096n

**Published:** 2012-05

**Authors:** EJC Dawe, A Saini, WN Bradley

**Affiliations:** Royal Surrey County Hospital NHS Foundation Trust,UK

## BACKGROUND

Distal femoral replacement is becoming an increasingly common procedure for patients with distal femoral fractures above a total knee arthroplasty and as revision knee arthroplasty becomes more complex.^1^ The femoral prosthesis must be excised with its accompanying distal bone fragment. Release of this fragment from soft tissue attachments at the knee may be time consuming as this fragment can be difficult to control. There is also a significant risk of vascular or neurological injury if dissection occurs away from the bone–soft tissue interface.

## TECHNIQUE

The senior author sites a corkscrew device transversely across the remaining bone of the femoral condyles inside the femoral prosthesis. The device is routinely used for removing the femoral head during hemiarthroplasty for fractured neck of femur. This provides excellent purchase on the fragment. The distal fragment may then be manipulated effectively to peel the distal femur out of its soft tissue envelope.

## DISCUSSION

Distal femoral replacement is a challenging procedure that may require extensive operative time.^2^ One review of such cases found a complication rate of between 25% and 75%.^3^ This technique provides a simple effective solution to a potentially time consuming step of a complex operation that may help avoid significant complications.

**Figure 1:**
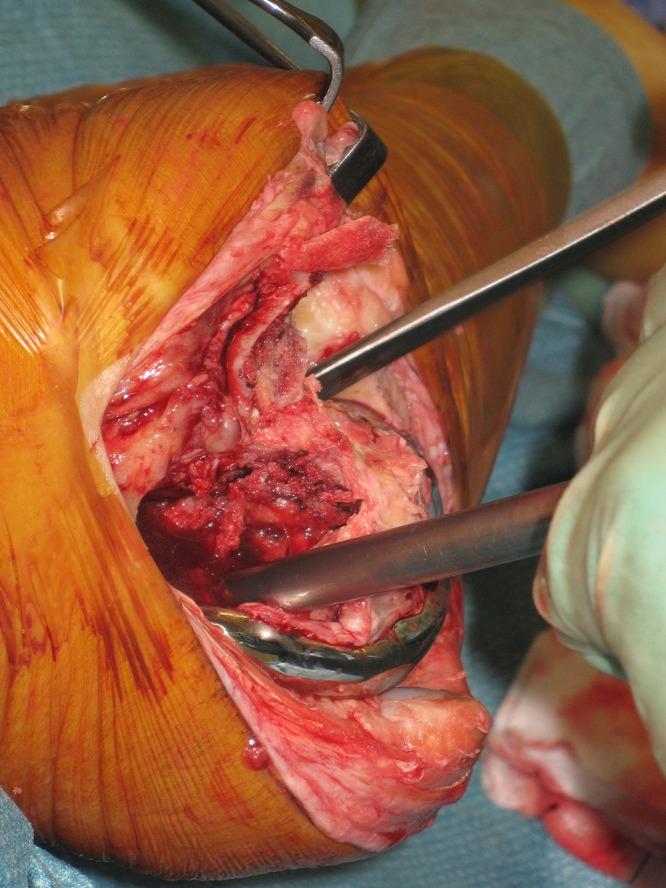
Dissection of soft tissue using the corkscrew to generate tension

**Figure 2:**
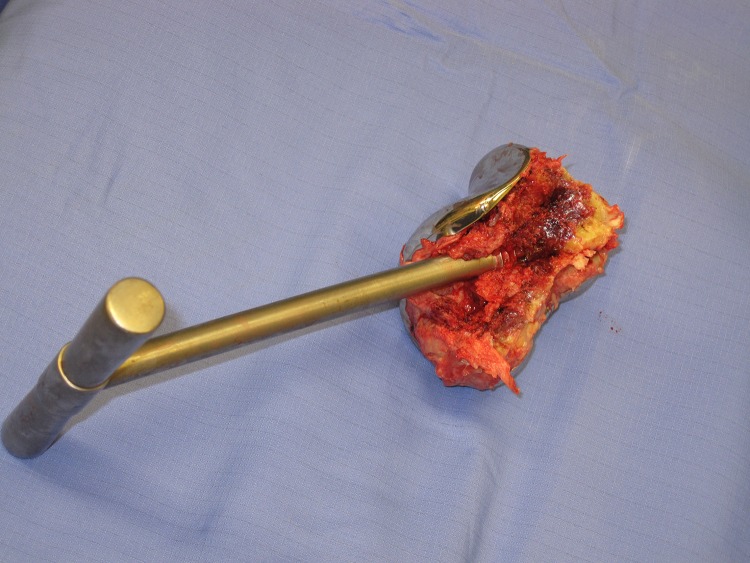
The fully excised femoral component showing how little soft tissue was excised along with the bony fragment using this technique
